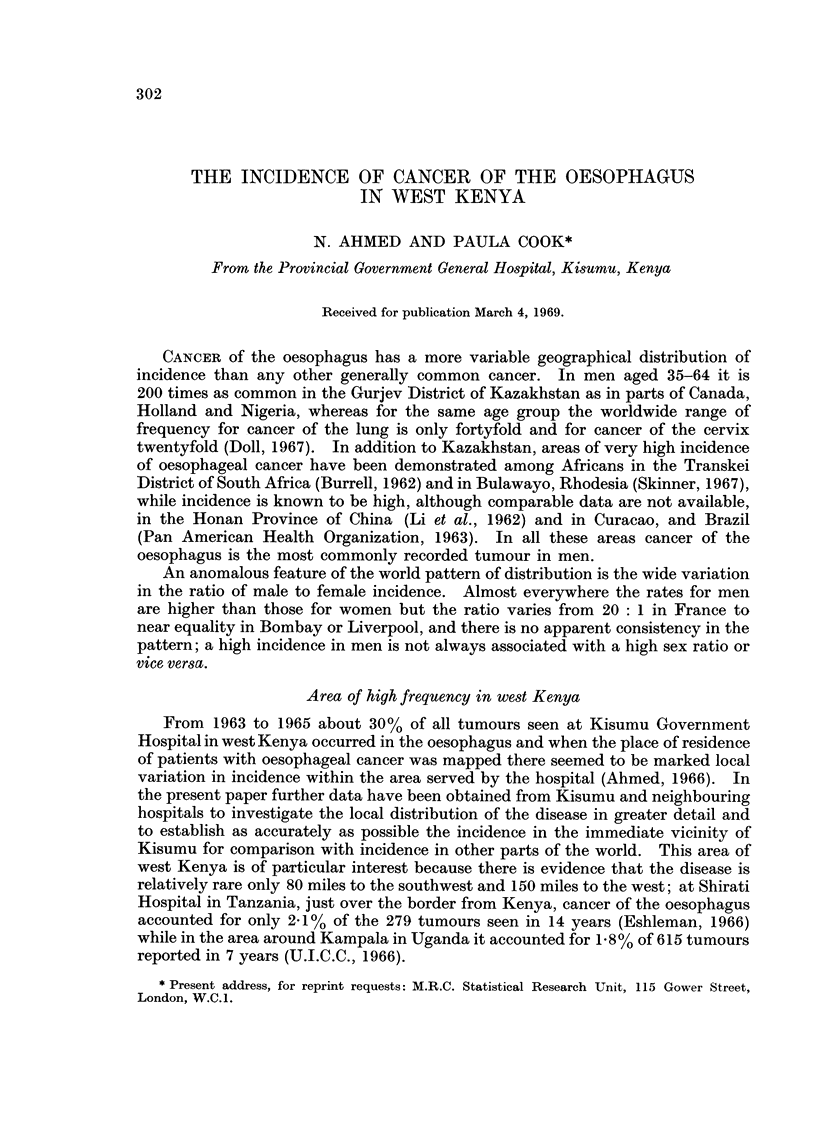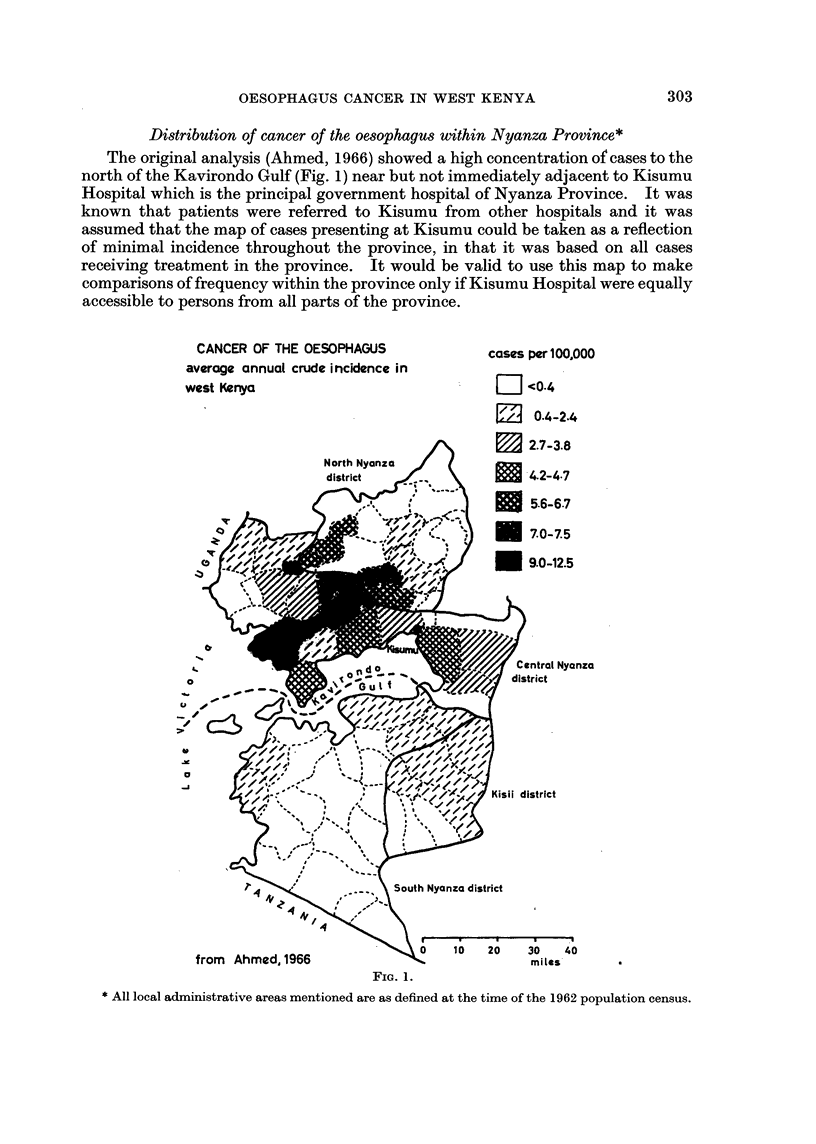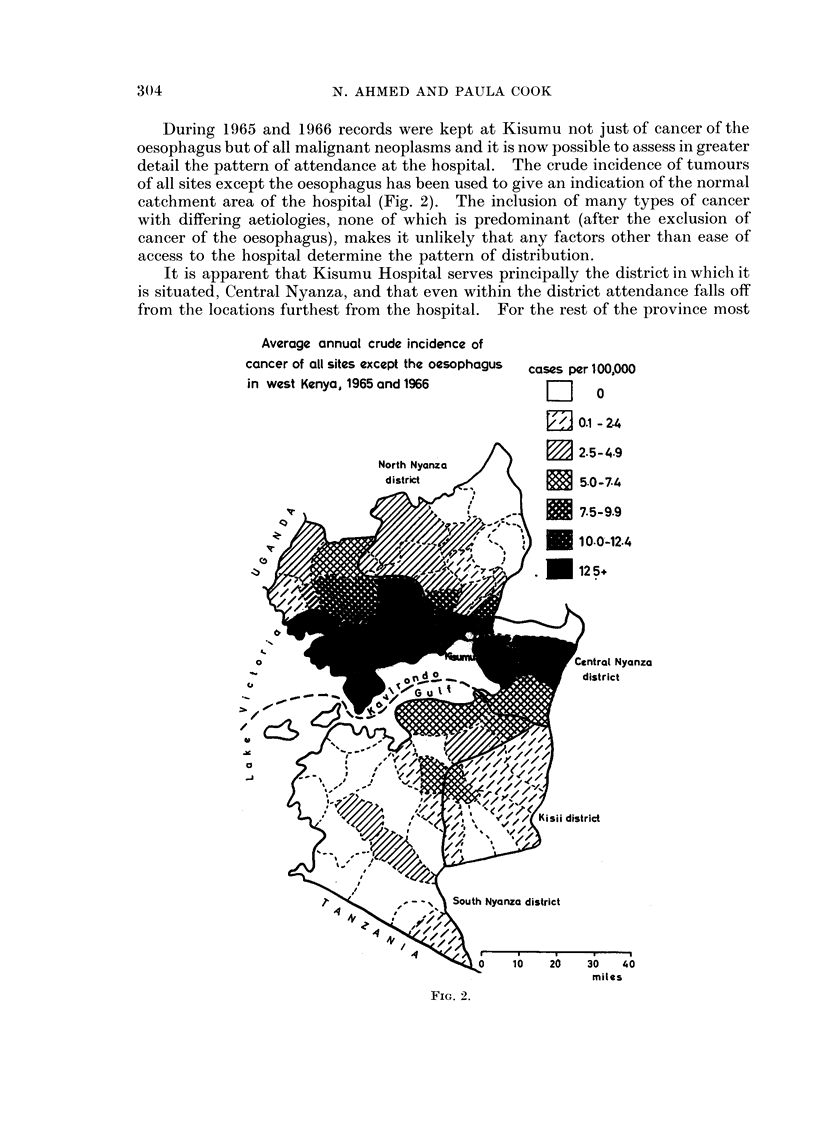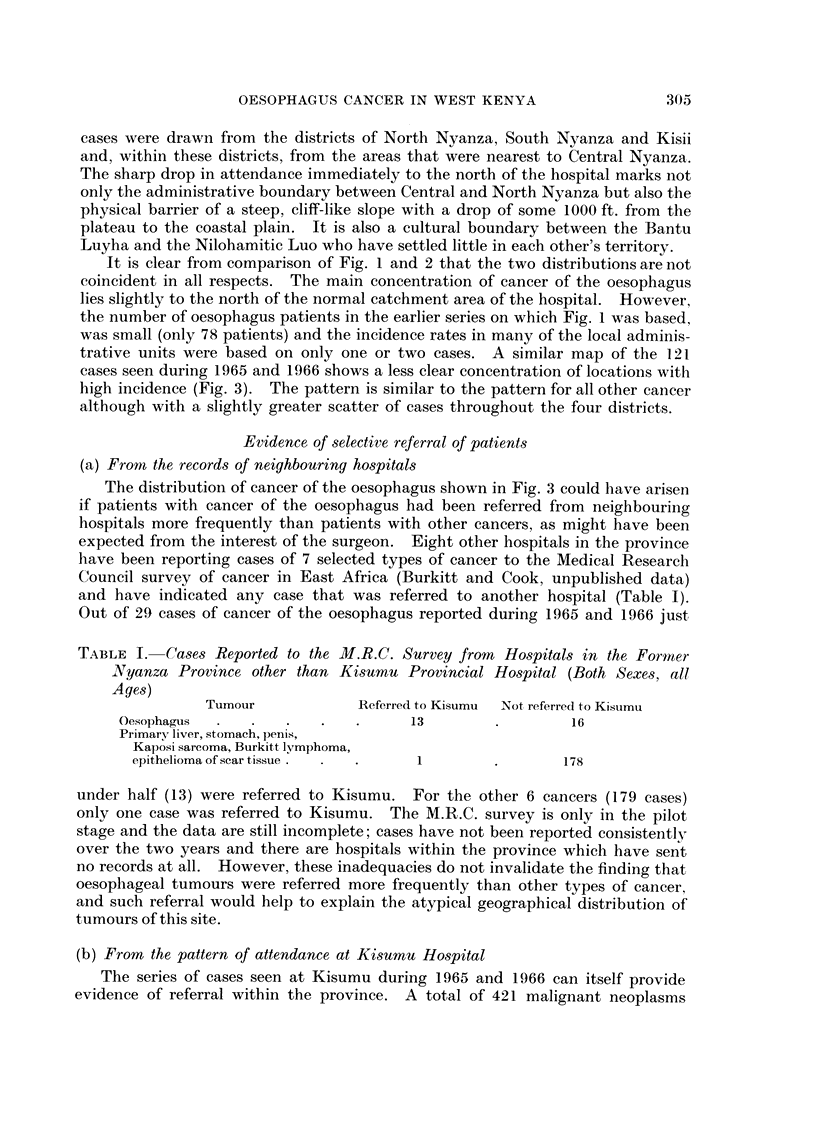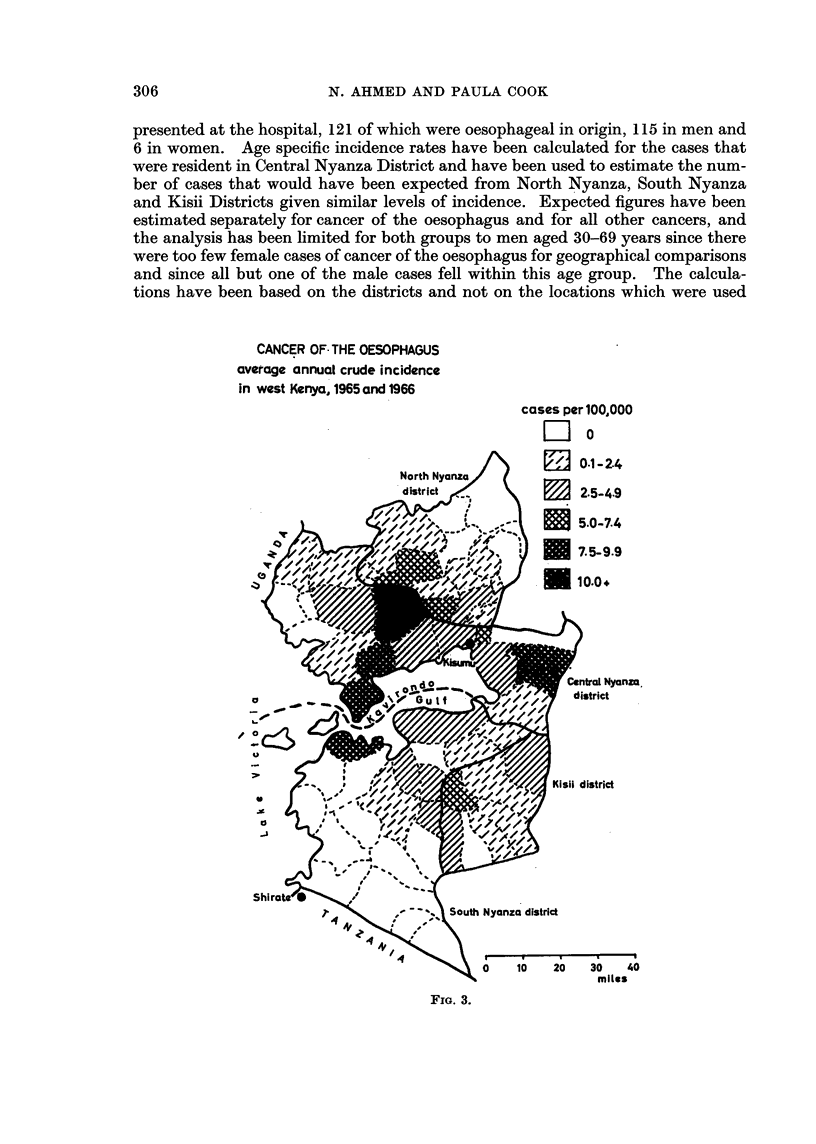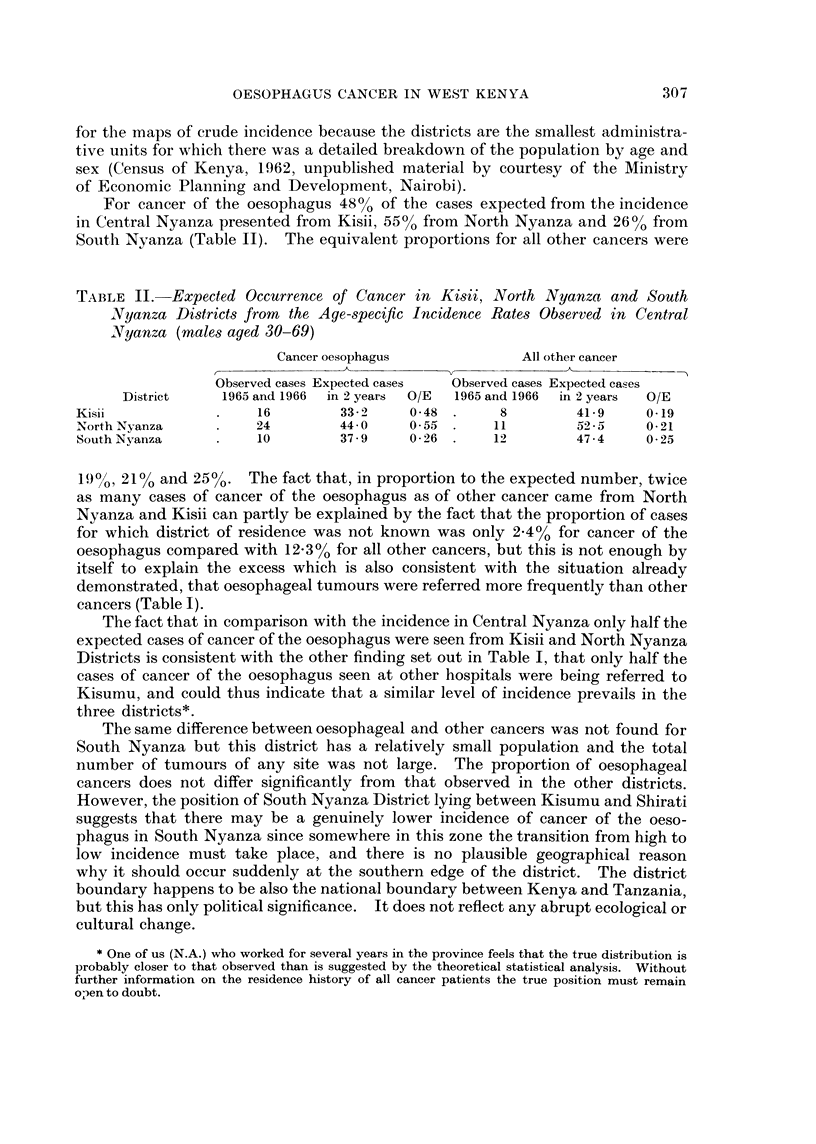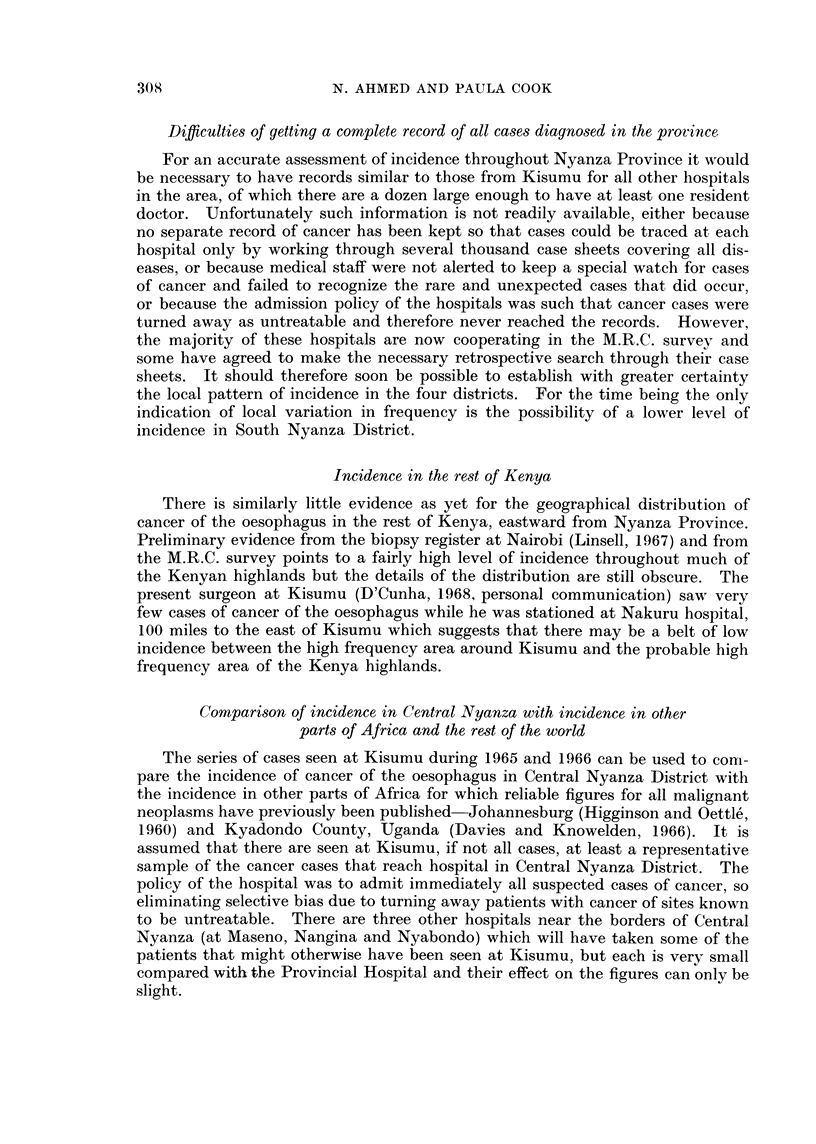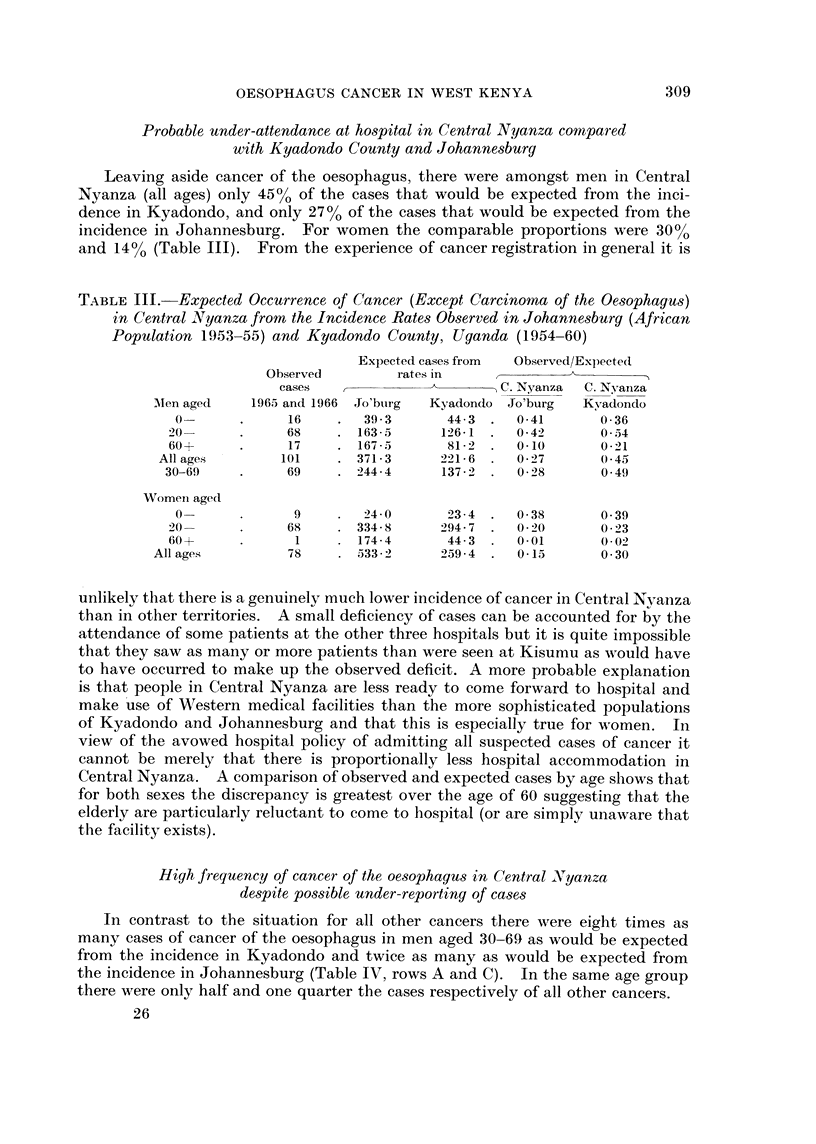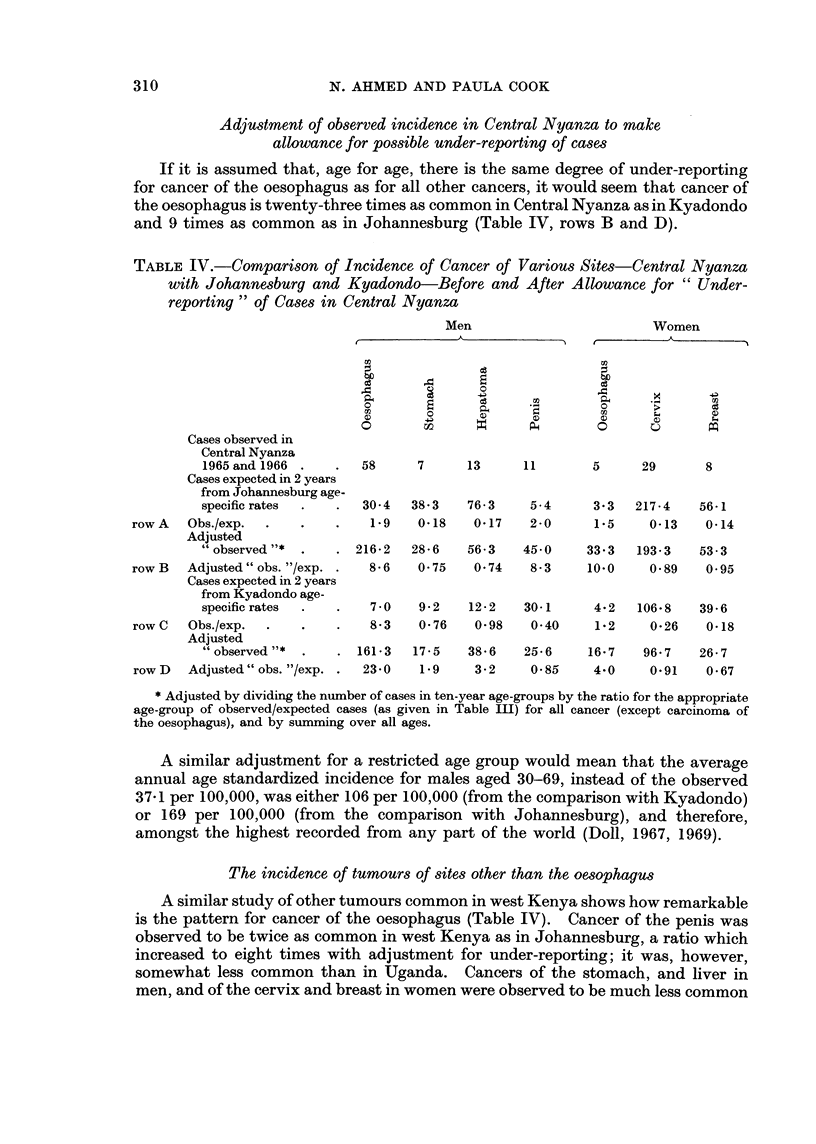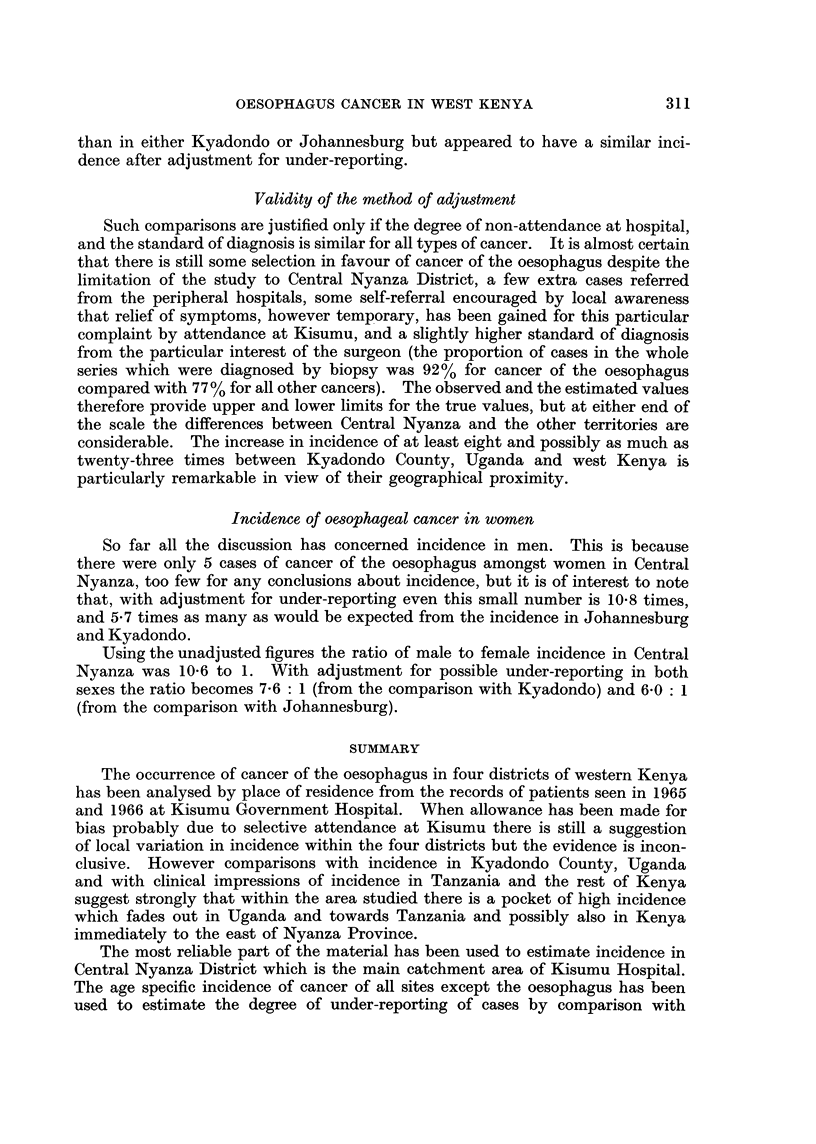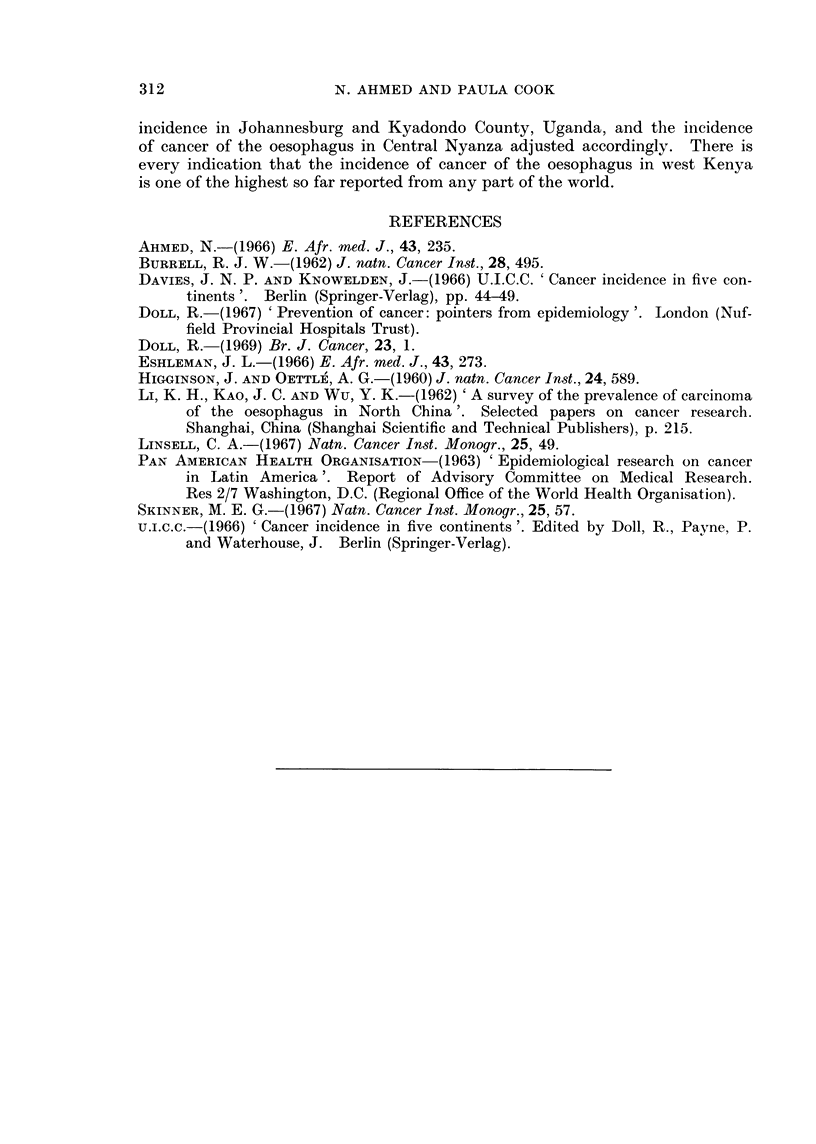# The incidence of cancer of the oesophagus in West Kenya.

**DOI:** 10.1038/bjc.1969.40

**Published:** 1969-06

**Authors:** N. Ahmed, P. Cook


### THE INCIDENCE OF CANCER OF THE OESOPHAGUS IN WEST KENYA

N. AHMED AND PAULA COOK*

*From the Provincial Government General Hospital, Kisumu, Kenya*

Received for publication March 4, 1969.

CANCER of the oesophagus has a more variable geographical distribution of incidence than any other generally common cancer. In men aged 35–64 it is 200 times as common in the Gurjev District of Kazakhstan as in parts of Canada, Holland and Nigeria, whereas for the same age group the worldwide range of frequency for cancer of the lung is only fortyfold and for cancer of the cervix twentyfold (Doll, 1967). In addition to Kazakhstan, areas of very high incidence of oesophageal cancer have been demonstrated among Africans in the Transkei District of South Africa (Burrell, 1962) and in Bulawayo, Rhodesia (Skinner, 1967), while incidence is known to be high, although comparable data are not available, in the Honan Province of China (Li *et al.*, 1962) and in Curacao, and Brazil (Pan American Health Organization, 1963). In all these areas cancer of the oesophagus is the most commonly recorded tumour in men.

An anomalous feature of the world pattern of distribution is the wide variation in the ratio of male to female incidence. Almost everywhere the rates for men are higher than those for women but the ratio varies from 20 : 1 in France to near equality in Bombay or Liverpool, and there is no apparent consistency in the pattern; a high incidence in men is not always associated with a high sex ratio or *vice versa.*

### *Area of high frequency in west Kenya*

From 1963 to 1965 about 30% of all tumours seen at Kisumu Government Hospital in west Kenya occurred in the oesophagus and when the place of residence of patients with oesophageal cancer was mapped there seemed to be marked local variation in incidence within the area served by the hospital (Ahmed, 1966). In the present paper further data have been obtained from Kisumu and neighbouring hospitals to investigate the local distribution of the disease in greater detail and to establish as accurately as possible the incidence in the immediate vicinity of Kisumu for comparison with incidence in other parts of the world. This area of west Kenya is of particular interest because there is evidence that the disease is relatively rare only 80 miles to the southwest and 150 miles to the west; at Shirati Hospital in Tanzania, just over the border from Kenya, cancer of the oesophagus accounted for only 2·1% of the 279 tumours seen in 14 years (Eshleman, 1966) while in the area around Kampala in Uganda it accounted for 1·8% of 615 tumours reported in 7 years (U.I.C.C., 1966).

* Present address, for reprint requests: M.R.C. Statistical Research Unit, 115 Gower Street, London, W.C.1.

*Distribution of cancer of the oesophagus within Nyanza Province**

The original analysis (Ahmed, 1966) showed a high concentration of cases to the north of the Kavirondo Gulf (Fig. 1) near but not immediately adjacent to Kisumu Hospital which is the principal government hospital of Nyanza Province. It was known that patients were referred to Kisumu from other hospitals and it was assumed that the map of cases presenting at Kisumu could be taken as a reflection of minimal incidence throughout the province, in that it was based on all cases receiving treatment in the province. It would be valid to use this map to make comparisons of frequency within the province only if Kisumu Hospital were equally accessible to persons from all parts of the province.

FIG. 1.

* All local administrative areas mentioned are as defined at the time of the 1962 population census.

During 1965 and 1966 records were kept at Kisumu not just of cancer of the oesophagus but of all malignant neoplasms and it is now possible to assess in greater detail the pattern of attendance at the hospital. The crude incidence of tumours of all sites except the oesophagus has been used to give an indication of the normal catchment area of the hospital (Fig. 2). The inclusion of many types of cancer with differing aetiologies, none of which is predominant (after the exclusion of cancer of the oesophagus), makes it unlikely that any factors other than ease of access to the hospital determine the pattern of distribution.

It is apparent that Kisumu Hospital serves principally the district in which it is situated, Central Nyanza, and that even within the district attendance falls off from the locations furthest from the hospital. For the rest of the province most

FIG. 2.

cases were drawn from the districts of North Nyanza, South Nyanza and Kisii and, within these districts, from the areas that were nearest to Central Nyanza. The sharp drop in attendance immediately to the north of the hospital marks not only the administrative boundary between Central and North Nyanza but also the physical barrier of a steep, cliff-like slope with a drop of some 1000 ft. from the plateau to the coastal plain. It is also a cultural boundary between the Bantu Luyha and the Nilohamitic Luo who have settled little in each other's territory.

It is clear from comparison of Fig. 1 and 2 that the two distributions are not coincident in all respects. The main concentration of cancer of the oesophagus lies slightly to the north of the normal catchment area of the hospital. However, the number of oesophagus patients in the earlier series on which Fig. 1 was based, was small (only 78 patients) and the incidence rates in many of the local administrative units were based on only one or two cases. A similar map of the 121 cases seen during 1965 and 1966 shows a less clear concentration of locations with high incidence (Fig. 3). The pattern is similar to the pattern for all other cancer although with a slightly greater scatter of cases throughout the four districts.

*Evidence of selective referral of patients*

(a) *From the records of neighbouring hospitals*

The distribution of cancer of the oesophagus shown in Fig. 3 could have arisen if patients with cancer of the oesophagus had been referred from neighbouring hospitals more frequently than patients with other cancers, as might have been expected from the interest of the surgeon. Eight other hospitals in the province have been reporting cases of 7 selected types of cancer to the Medical Research Council survey of cancer in East Africa (Burkitt and Cook, unpublished data) and have indicated any case that was referred to another hospital (Table I). Out of 29 cases of cancer of the oesophagus reported during 1965 and 1966 just

TABLE I.—*Cases Reported to the M.R.C. Survey from Hospitals in the Former Nyanza Province other than Kisumu Provincial Hospital (Both Sexes, all Ages)*

| Tumour | Referred to Kisumu | Not referred to Kisumu |
|---|---|---|
| Oesophagus . . . . . | 13 | 16 |
| Primary liver, stomach, penis, Kaposi sarcoma, Burkitt lymphoma, epithelioma of scar tissue . . . | 1 | 178 |

under half (13) were referred to Kisumu. For the other 6 cancers (179 cases) only one case was referred to Kisumu. The M.R.C. survey is only in the pilot stage and the data are still incomplete; cases have not been reported consistently over the two years and there are hospitals within the province which have sent no records at all. However, these inadequacies do not invalidate the finding that oesophageal tumours were referred more frequently than other types of cancer, and such referral would help to explain the atypical geographical distribution of tumours of this site.

(b) *From the pattern of attendance at Kisumu Hospital*

The series of cases seen at Kisumu during 1965 and 1966 can itself provide evidence of referral within the province. A total of 421 malignant neoplasms presented at the hospital, 121 of which were oesophageal in origin, 115 in men and 6 in women. Age specific incidence rates have been calculated for the cases that were resident in Central Nyanza District and have been used to estimate the number of cases that would have been expected from North Nyanza, South Nyanza and Kisii Districts given similar levels of incidence. Expected figures have been estimated separately for cancer of the oesophagus and for all other cancers, and the analysis has been limited for both groups to men aged 30–69 years since there were too few female cases of cancer of the oesophagus for geographical comparisons and since all but one of the male cases fell within this age group. The calculations have been based on the districts and not on the locations which were used

FIG. 3.

for the maps of crude incidence because the districts are the smallest administrative units for which there was a detailed breakdown of the population by age and sex (Census of Kenya, 1962, unpublished material by courtesy of the Ministry of Economic Planning and Development, Nairobi).

For cancer of the oesophagus 48% of the cases expected from the incidence in Central Nyanza presented from Kisii, 55% from North Nyanza and 26% from South Nyanza (Table II). The equivalent proportions for all other cancers were 19%, 21% and 25%. The fact that, in proportion to the expected number, twice as many cases of cancer of the oesophagus as of other cancer came from North Nyanza and Kisii can partly be explained by the fact that the proportion of cases for which district of residence was not known was only 2·4% for cancer of the oesophagus compared with 12·3% for all other cancers, but this is not enough by itself to explain the excess which is also consistent with the situation already demonstrated, that oesophageal tumours were referred more frequently than other cancers (Table I).

TABLE II.—*Expected Occurrence of Cancer in Kisii, North Nyanza and South Nyanza Districts from the Age-specific Incidence Rates Observed in Central Nyanza (males aged 30–69)*

| | Cancer oesophagus | | | All other cancer | | |
|---|---|---|---|---|---|---|
| District | Observed cases 1965 and 1966 | Expected cases in 2 years | O/E | Observed cases 1965 and 1966 | Expected cases in 2 years | O/E |
| Kisii | 16 | 33·2 | 0·48 | 8 | 41·9 | 0·19 |
| North Nyanza | 24 | 44·0 | 0·55 | 11 | 52·5 | 0·21 |
| South Nyanza | 10 | 37·9 | 0·26 | 12 | 47·4 | 0·25 |

The fact that in comparison with the incidence in Central Nyanza only half the expected cases of cancer of the oesophagus were seen from Kisii and North Nyanza Districts is consistent with the other finding set out in Table I, that only half the cases of cancer of the oesophagus seen at other hospitals were being referred to Kisumu, and could thus indicate that a similar level of incidence prevails in the three districts*.

The same difference between oesophageal and other cancers was not found for South Nyanza but this district has a relatively small population and the total number of tumours of any site was not large. The proportion of oesophageal cancers does not differ significantly from that observed in the other districts. However, the position of South Nyanza District lying between Kisumu and Shirati suggests that there may be a genuinely lower incidence of cancer of the oesophagus in South Nyanza since somewhere in this zone the transition from high to low incidence must take place, and there is no plausible geographical reason why it should occur suddenly at the southern edge of the district. The district boundary happens to be also the national boundary between Kenya and Tanzania, but this has only political significance. It does not reflect any abrupt ecological or cultural change.

* One of us (N.A.) who worked for several years in the province feels that the true distribution is probably closer to that observed than is suggested by the theoretical statistical analysis. Without further information on the residence history of all cancer patients the true position must remain open to doubt.

*Difficulties of getting a complete record of all cases diagnosed in the province*

For an accurate assessment of incidence throughout Nyanza Province it would be necessary to have records similar to those from Kisumu for all other hospitals in the area, of which there are a dozen large enough to have at least one resident doctor. Unfortunately such information is not readily available, either because no separate record of cancer has been kept so that cases could be traced at each hospital only by working through several thousand case sheets covering all diseases, or because medical staff were not alerted to keep a special watch for cases of cancer and failed to recognize the rare and unexpected cases that did occur, or because the admission policy of the hospitals was such that cancer cases were turned away as untreatable and therefore never reached the records. However, the majority of these hospitals are now cooperating in the M.R.C. survey and some have agreed to make the necessary retrospective search through their case sheets. It should therefore soon be possible to establish with greater certainty the local pattern of incidence in the four districts. For the time being the only indication of local variation in frequency is the possibility of a lower level of incidence in South Nyanza District.

*Incidence in the rest of Kenya*

There is similarly little evidence as yet for the geographical distribution of cancer of the oesophagus in the rest of Kenya, eastward from Nyanza Province. Preliminary evidence from the biopsy register at Nairobi (Linsell, 1967) and from the M.R.C. survey points to a fairly high level of incidence throughout much of the Kenyan highlands but the details of the distribution are still obscure. The present surgeon at Kisumu (D'Cunha, 1968, personal communication) saw very few cases of cancer of the oesophagus while he was stationed at Nakuru hospital, 100 miles to the east of Kisumu which suggests that there may be a belt of low incidence between the high frequency area around Kisumu and the probable high frequency area of the Kenya highlands.

*Comparison of incidence in Central Nyanza with incidence in other parts of Africa and the rest of the world*

The series of cases seen at Kisumu during 1965 and 1966 can be used to compare the incidence of cancer of the oesophagus in Central Nyanza District with the incidence in other parts of Africa for which reliable figures for all malignant neoplasms have previously been published—Johannesburg (Higginson and Oettlé, 1960) and Kyadondo County, Uganda (Davies and Knowelden, 1966). It is assumed that there are seen at Kisumu, if not all cases, at least a representative sample of the cancer cases that reach hospital in Central Nyanza District. The policy of the hospital was to admit immediately all suspected cases of cancer, so eliminating selective bias due to turning away patients with cancer of sites known to be untreatable. There are three other hospitals near the borders of Central Nyanza (at Maseno, Nangina and Nyabondo) which will have taken some of the patients that might otherwise have been seen at Kisumu, but each is very small compared with the Provincial Hospital and their effect on the figures can only be slight.

*Probable under-attendance at hospital in Central Nyanza compared with Kyadondo County and Johannesburg*

Leaving aside cancer of the oesophagus, there were amongst men in Central Nyanza (all ages) only 45% of the cases that would be expected from the incidence in Kyadondo, and only 27% of the cases that would be expected from the incidence in Johannesburg. For women the comparable proportions were 30% and 14% (Table III). From the experience of cancer registration in general it is

TABLE III.—*Expected Occurrence of Cancer (Except Carcinoma of the Oesophagus) in Central Nyanza from the Incidence Rates Observed in Johannesburg (African Population* 1953–55) *and Kyadondo County, Uganda* (1954–60)

| | Observed cases | Expected cases from rates in | | Observed/Expected | |
|---|---|---|---|---|---|
| Men aged | 1965 and 1966 | Jo'burg | Kyadondo | C. Nyanza / Jo'burg | C. Nyanza / Kyadondo |
| 0– | 16 | 39·3 | 44·3 | 0·41 | 0·36 |
| 20– | 68 | 163·5 | 126·1 | 0·42 | 0·54 |
| 60+ | 17 | 167·5 | 81·2 | 0·10 | 0·21 |
| All ages | 101 | 371·3 | 221·6 | 0·27 | 0·45 |
| 30–69 | 69 | 244·4 | 137·2 | 0·28 | 0·49 |
| Women aged | | | | | |
| 0– | 9 | 24·0 | 23·4 | 0·38 | 0·39 |
| 20– | 68 | 334·8 | 294·7 | 0·20 | 0·23 |
| 60+ | 1 | 174·4 | 44·3 | 0·01 | 0·02 |
| All ages | 78 | 533·2 | 259·4 | 0·15 | 0·30 |

unlikely that there is a genuinely much lower incidence of cancer in Central Nyanza than in other territories. A small deficiency of cases can be accounted for by the attendance of some patients at the other three hospitals but it is quite impossible that they saw as many or more patients than were seen at Kisumu as would have to have occurred to make up the observed deficit. A more probable explanation is that people in Central Nyanza are less ready to come forward to hospital and make use of Western medical facilities than the more sophisticated populations of Kyadondo and Johannesburg and that this is especially true for women. In view of the avowed hospital policy of admitting all suspected cases of cancer it cannot be merely that there is proportionally less hospital accommodation in Central Nyanza. A comparison of observed and expected cases by age shows that for both sexes the discrepancy is greatest over the age of 60 suggesting that the elderly are particularly reluctant to come to hospital (or are simply unaware that the facility exists).

*High frequency of cancer of the oesophagus in Central Nyanza despite possible under-reporting of cases*

In contrast to the situation for all other cancers there were eight times as many cases of cancer of the oesophagus in men aged 30–69 as would be expected from the incidence in Kyadondo and twice as many as would be expected from the incidence in Johannesburg (Table IV, rows A and C). In the same age group there were only half and one quarter the cases respectively of all other cancers.

### *Adjustment of observed incidence in Central Nyanza to make allowance for possible under-reporting of cases*

If it is assumed that, age for age, there is the same degree of under-reporting for cancer of the oesophagus as for all other cancers, it would seem that cancer of the oesophagus is twenty-three times as common in Central Nyanza as in Kyadondo and 9 times as common as in Johannesburg (Table IV, rows B and D).

TABLE IV.—*Comparison of Incidence of Cancer of Various Sites—Central Nyanza with Johannesburg and Kyadondo—Before and After Allowance for "Under-reporting" of Cases in Central Nyanza*

| | | Men | | | | Women | | |
|---|---|---|---|---|---|---|---|---|
| | | Oesophagus | Stomach | Hepatoma | Penis | Oesophagus | Cervix | Breast |
| | Cases observed in Central Nyanza 1965 and 1966 . . | 58 | 7 | 13 | 11 | 5 | 29 | 8 |
| | Cases expected in 2 years from Johannesburg age-specific rates . . | 30·4 | 38·3 | 76·3 | 5·4 | 3·3 | 217·4 | 56·1 |
| row A | Obs./exp. . . . | 1·9 | 0·18 | 0·17 | 2·0 | 1·5 | 0·13 | 0·14 |
| | Adjusted "observed"* . . | 216·2 | 28·6 | 56·3 | 45·0 | 33·3 | 193·3 | 53·3 |
| row B | Adjusted "obs."/exp. . | 8·6 | 0·75 | 0·74 | 8·3 | 10·0 | 0·89 | 0·95 |
| | Cases expected in 2 years from Kyadondo age-specific rates . . | 7·0 | 9·2 | 12·2 | 30·1 | 4·2 | 106·8 | 39·6 |
| row C | Obs./exp. . . . | 8·3 | 0·76 | 0·98 | 0·40 | 1·2 | 0·26 | 0·18 |
| | Adjusted "observed"* . . | 161·3 | 17·5 | 38·6 | 25·6 | 16·7 | 96·7 | 26·7 |
| row D | Adjusted "obs."/exp. . | 23·0 | 1·9 | 3·2 | 0·85 | 4·0 | 0·91 | 0·67 |

* Adjusted by dividing the number of cases in ten-year age-groups by the ratio for the appropriate age-group of observed/expected cases (as given in Table III) for all cancer (except carcinoma of the oesophagus), and by summing over all ages.

A similar adjustment for a restricted age group would mean that the average annual age standardized incidence for males aged 30–69, instead of the observed 37·1 per 100,000, was either 106 per 100,000 (from the comparison with Kyadondo) or 169 per 100,000 (from the comparison with Johannesburg), and therefore, amongst the highest recorded from any part of the world (Doll, 1967, 1969).

### *The incidence of tumours of sites other than the oesophagus*

A similar study of other tumours common in west Kenya shows how remarkable is the pattern for cancer of the oesophagus (Table IV). Cancer of the penis was observed to be twice as common in west Kenya as in Johannesburg, a ratio which increased to eight times with adjustment for under-reporting; it was, however, somewhat less common than in Uganda. Cancers of the stomach, and liver in men, and of the cervix and breast in women were observed to be much less common than in either Kyadondo or Johannesburg but appeared to have a similar incidence after adjustment for under-reporting.

### *Validity of the method of adjustment*

Such comparisons are justified only if the degree of non-attendance at hospital, and the standard of diagnosis is similar for all types of cancer. It is almost certain that there is still some selection in favour of cancer of the oesophagus despite the limitation of the study to Central Nyanza District, a few extra cases referred from the peripheral hospitals, some self-referral encouraged by local awareness that relief of symptoms, however temporary, has been gained for this particular complaint by attendance at Kisumu, and a slightly higher standard of diagnosis from the particular interest of the surgeon (the proportion of cases in the whole series which were diagnosed by biopsy was 92% for cancer of the oesophagus compared with 77% for all other cancers). The observed and the estimated values therefore provide upper and lower limits for the true values, but at either end of the scale the differences between Central Nyanza and the other territories are considerable. The increase in incidence of at least eight and possibly as much as twenty-three times between Kyadondo County, Uganda and west Kenya is particularly remarkable in view of their geographical proximity.

### *Incidence of oesophageal cancer in women*

So far all the discussion has concerned incidence in men. This is because there were only 5 cases of cancer of the oesophagus amongst women in Central Nyanza, too few for any conclusions about incidence, but it is of interest to note that, with adjustment for under-reporting even this small number is 10·8 times, and 5·7 times as many as would be expected from the incidence in Johannesburg and Kyadondo.

Using the unadjusted figures the ratio of male to female incidence in Central Nyanza was 10·6 to 1. With adjustment for possible under-reporting in both sexes the ratio becomes 7·6 : 1 (from the comparison with Kyadondo) and 6·0 : 1 (from the comparison with Johannesburg).

### SUMMARY

The occurrence of cancer of the oesophagus in four districts of western Kenya has been analysed by place of residence from the records of patients seen in 1965 and 1966 at Kisumu Government Hospital. When allowance has been made for bias probably due to selective attendance at Kisumu there is still a suggestion of local variation in incidence within the four districts but the evidence is inconclusive. However comparisons with incidence in Kyadondo County, Uganda and with clinical impressions of incidence in Tanzania and the rest of Kenya suggest strongly that within the area studied there is a pocket of high incidence which fades out in Uganda and towards Tanzania and possibly also in Kenya immediately to the east of Nyanza Province.

The most reliable part of the material has been used to estimate incidence in Central Nyanza District which is the main catchment area of Kisumu Hospital. The age specific incidence of cancer of all sites except the oesophagus has been used to estimate the degree of under-reporting of cases by comparison with incidence in Johannesburg and Kyadondo County, Uganda, and the incidence of cancer of the oesophagus in Central Nyanza adjusted accordingly. There is every indication that the incidence of cancer of the oesophagus in west Kenya is one of the highest so far reported from any part of the world.

### REFERENCES

AHMED, N.—(1966) *E. Afr. med. J.*, **43**, 235.
BURRELL, R. J. W.—(1962) *J. natn. Cancer Inst.*, **28**, 495.
DAVIES, J. N. P. AND KNOWELDEN, J.—(1966) U.I.C.C. ' Cancer incidence in five continents '. Berlin (Springer-Verlag), pp. 44–49.
DOLL, R.—(1967) ' Prevention of cancer: pointers from epidemiology '. London (Nuffield Provincial Hospitals Trust).
DOLL, R.—(1969) *Br. J. Cancer*, **23**, 1.
ESHLEMAN, J. L.—(1966) *E. Afr. med. J.*, **43**, 273.
HIGGINSON, J. AND OETTLÉ, A. G.—(1960) *J. natn. Cancer Inst.*, **24**, 589.
LI, K. H., KAO, J. C. AND WU, Y. K.—(1962) ' A survey of the prevalence of carcinoma of the oesophagus in North China '. Selected papers on cancer research. Shanghai, China (Shanghai Scientific and Technical Publishers), p. 215.
LINSELL, C. A.—(1967) *Natn. Cancer Inst. Monogr.*, **25**, 49.
PAN AMERICAN HEALTH ORGANISATION—(1963) ' Epidemiological research on cancer in Latin America '. Report of Advisory Committee on Medical Research. Res 2/7 Washington, D.C. (Regional Office of the World Health Organisation).
SKINNER, M. E. G.—(1967) *Natn. Cancer Inst. Monogr.*, **25**, 57.
U.I.C.C.—(1966) ' Cancer incidence in five continents '. Edited by Doll, R., Payne, P. and Waterhouse, J. Berlin (Springer-Verlag).